# Plant-expressed pyocins for control of *Pseudomonas aeruginosa*

**DOI:** 10.1371/journal.pone.0185782

**Published:** 2017-10-03

**Authors:** Šarūnas Paškevičius, Urtė Starkevič, Audrius Misiūnas, Astra Vitkauskienė, Yuri Gleba, Aušra Ražanskienė

**Affiliations:** 1 Nomads UAB, Geležinio vilko 29A, Vilnius, Lithuania; 2 Vilnius University, Institute of Biotechnology, Saulėtekio al. 7, Vilnius, Lithuania; 3 Lithuanian University of Health Sciences, A. Mickevičiaus g. 9, Kaunas, Lithuania; 4 Nomad Bioscience GmbH, Biozentrum Halle, Weinbergweg 22, Halle (Saale), Germany; Indiana University, UNITED STATES

## Abstract

The emergence, persistence and spread of antibiotic-resistant human pathogenic bacteria heralds a growing global health crisis. Drug-resistant strains of gram-negative bacteria, such as *Pseudomonas aeruginosa*, are especially dangerous and the medical and economic burden they impose underscore the critical need for finding new antimicrobials. Recent studies have demonstrated that plant-expressed bacteriocins of the colicins family can be efficient antibacterials against all major enteropathogenic strains of *E*. *coli*. We extended our studies of colicin-like bacteriocins to pyocins, which are produced by strains of *P*. *aeruginosa* for ecological advantage against other strains of the same species. Using a plant-based transient expression system, we expressed six different pyocins, namely S5, PaeM, L1, L2, L3 and one new pyocin, PaeM4, and purified them to homogeneity. Among these pyocins, PaeM4 demonstrated the broadest spectrum of activity by controlling 53 of 100 tested clinical isolates of *P*. *aeruginosa*. The activity of plant-made pyocins was confirmed in the agar drop, liquid culture susceptibility and biofilm assays, and in the *Galleria mellonella* animal infection model.

## Introduction

*Pseudomonas aeruginosa* is a ubiquitous Gram-negative, rod-shaped, asporogenous, monoflagellated bacterium belonging to the class Gammaproteobacteria, and is characterized in part by its high nutritional versatility. Consequently, *P*. *aeruginosa* can persist in diverse environments including water and the rhizosphere, as well as in the human body. *P*. *aeruginosa* is an opportunistic pathogen and can cause diseases in plants and animals, including humans. In the latter, it can cause life-threatening chronic infections (pneumonia, ventilator-associated pneumonia, catheter-related infections, burn wound infections, and sepsis) especially in patients with a weakened immune system. This bacterium is one of the six pathogens causing hospital ESKAPE (*Enterococcus faecium*, *Staphylococcus aureus*, *Klebsiella pneumoniae*, *Acinetobacter baumannii*, *Pseudomonas aeruginosa*, *Enterobacter*) infections, which readily develop resistance to antibiotics [1]. Recently, the WHO published a list of bacteria for which new antibiotics are urgently needed, and carbapenem-resistant *Pseudomonas aeruginosa* was declared a problem of critical importance (http://www.who.int/mediacentre/news/releases/2017/bacteria-antibiotics-needed/en/).

In addition to nosocomial infections, *P*. *aeruginosa* is very dangerous for cystic fibrosis sufferers. Cystic fibrosis (CF), or mucoviscidosis, is an autosomal recessive genetic disease most common in Northern Europe and USA. The disease is caused by a mutation in the gene encoding the CFTR protein. CFTR is a membrane permeability regulator, and its malfunction causes the production of abnormally viscous and sticky mucus, which accumulates instead of leaving the site of production. The lungs of patients with CF become prone to infections. When *P*. *aeruginosa* colonizes the lungs of CF patients and chemotherapy is unsuccessful, the infection becomes chronic and lung function deteriorates irreversibly.

Ever increasing pathogen drug resistance is a global problem. The optimism that followed the discovery of antibiotics became short-lived once it became known that microbes could develop resistance to all classes of therapeutics that were once effective. The race was on to keep ahead of the evolving resistance problem. Unfortunately, in recent decades antibiotic discovery programs have yielded diminishing returns, with fewer antibiotics being registered each year. The traditional approach of screening large libraries of synthetic compounds, and to a lesser extent natural-compounds, has produced initially interesting leads that unfortunately have not always passed secondary screenings for safety in human cells. Moreover, many compounds that pass the initial screenings fail in subsequent stages of development because of their inability to enter bacterial cells. Solving the problem of membrane permeability has become one of the most critical challenges to finding new safe and effective antibacterials. Progress in this area will be greatly enhanced once permeability can be modeled and predicted [2]. As a result of these collective challenges, only a small fraction of potential novel drugs ever reaches clinical trials. The success rate of antimicrobial drugs in clinical studies is less than 20%; thus, we can expect to see only one or two new drugs against Gram-negative pathogens in the near future, possibly without knowing whether they will be effective against *P*. *aeruginosa* in a clinical setting.

In such context, the development of a new generation of antimicrobial substances is needed and nature may again provide a source of solutions. Bacteriocins are the weapons naturally produced by various genera of bacteria to compete for ecological niches. *P*. *aeruginosa* strains compete with each other by secreting antibacterial proteins called pyocins. This heterogeneous group of proteins includes deoxyribonucleases, ribonucleases, membrane pore-formers, peptidoglycan synthesis-blockers, lectin-like proteins, and bacteriophage tail-like protein complexes, among the range of specificities studied [3,4]. Applicability of some pyocins to treat *P*. *aeruginosa* infections has been demonstrated in animal models [5–7].

We expressed pyocins of all listed types in a highly efficient plant-based transient gene expression system [8,9]. This system enables expression of a wide-range of recombinant proteins, including some bacteriocins that cannot be expressed in bacteria due to host toxicity. Plant-based systems often yield recombinant proteins with purification and cost advantages over their microbial and mammalian cell culture counterparts, because the bulk product does not contain endotoxin or carries the risk of adventitious agents that require laborious removal and stringent safety checks. Recently, we used this system to produce in plants several colicins to be applied as food antimicrobials to control contamination by enterohemorrhagic *E*. *coli* [10]. The plant-made colicins have received marketing allowance through the US Food and Drug Administration's (FDA) GRAS (Generally Recognized As Safe) regulatory review process. In the current study, we used plant-based expression to produce fully functional pyocins and evaluated their activity against several strains of *P*. *aeruginosa*. We observed that pyocins that act through inhibition of cell wall synthesis, induce pore formation, or exhibit lectin-like binding, target the widest spectrum of pathogenic *P*. *aeruginosa* strains. We describe herein the expression, purification and characterization of pyocins S5, PaeM, three lectin-like pyocins (L1, L2 and L3) and one new pyocin, PaeM4. We demonstrate the possibility of using these pyocins alone or in cocktails to combat *Pseudomonas aeruginosa* based on results obtained *in vitro* and in the *Galleria mellonella* animal infection model.

## Methods

### Bacterial strains and cultures

Unless otherwise stated, *P*. *aeruginosa* strains were prepared by culturing in Lysogeny Broth (LB) medium (*Roth*) or Casamino Acids (0.5% Bacto^TM^ Casamino acids, 5.2 mM K_2_HPO_4_, 1 mM MgSO_4_) medium (BD Bacto) at 37°C with shaking (200 rpm); overnight cultures were prepared by inoculation from frozen stocks.

*P*. *aeruginosa* strains used in experiments are described in Supplementary information (S1 Table).

### Construction of pyocin expression vectors

**Fig 1:**
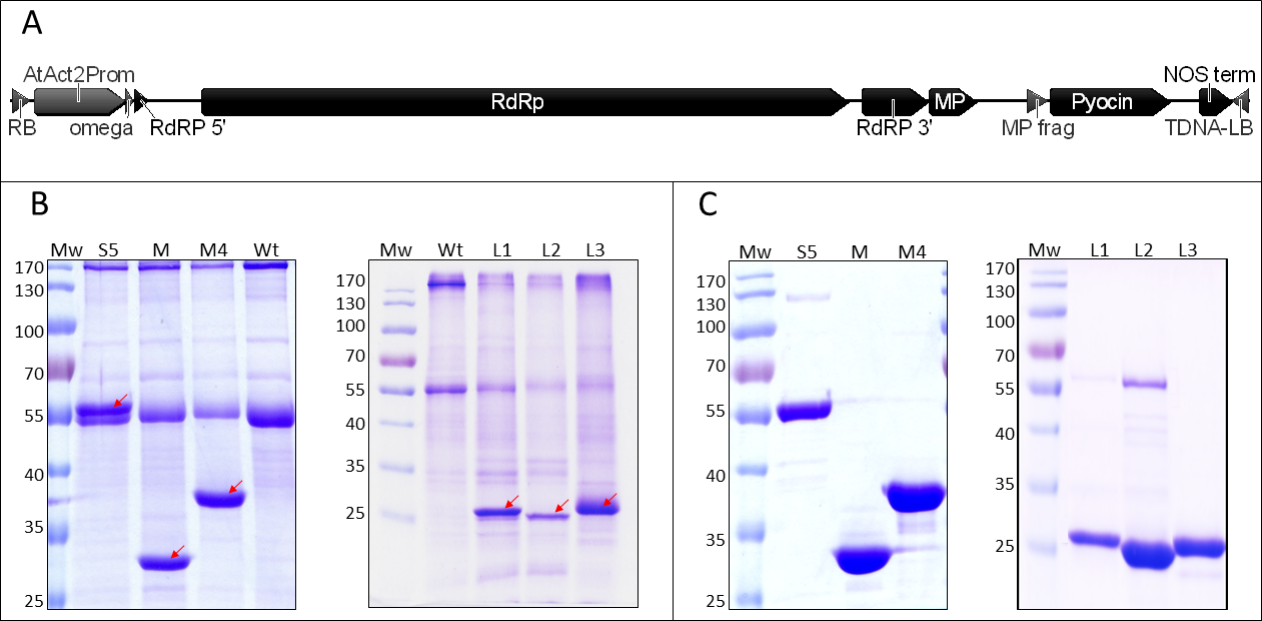
Pyocin expression in plants. **A–schematic presentation of T-DNA region with pyocin expression cassette.** RB–right T-DNA border, AtAct23Prom–*A*. *thaliana* actin promoter, RdRp–RNA-dependent RNA polymerase, MP–truncated TMV movement protein, LB–left T-DNA border. **B–expression of pyocins in *N*. *benthamiana* leaves.** Plant material (50 mg) was harvested at 5 or 7 days post spraying (dps) (pooled samples of three leaves—pyocins S5, PaeM, PaeM4, L1, L2 at 5 dps, L3 at 7 dps), ground in liquid nitrogen, extracted with 50 mM Tris-HCl, 150 mM NaCl (pH 7.0) (S5, PaeM and PaeM4) or 50 mM HEPES, 10 mM CH_3_COOK, 5 mM Mg(CH_3_COO)_2_, 2 mM DTT, 1 mM EDTA, pH 5.0 (L1, L2, L3) and denatured at 98°C for 10 min. Solutions containing 5 μg of protein were resolved in 12% polyacrylamide gel for Coomassie staining. Mw–PageRuler Prestained protein ladder (ThermoFisher Scientific Baltics), Wt–crude extract of non—sprayed *N*. *benthamiana* leaves, S5, M, M4, L1, L2, L3 –extracts of *N*. *benthamiana* leaves, sprayed with pyocin expression constructs (pyocin S5, PaeM, PaeM4 and lectin–like pyocins L1, L2, L3). Bands corresponding to recombinant pyocins are marked by arrows. **C–purified pyocins**. Pyocins were purified by two-step chromatography as described in Purification section of Methods, and resolved in 12% polyacrylamide gel for Coomassie staining.

The PaeM4, PaeM_JJ692_, pyocins S5, L1, L2 and L3 coding sequences (NCBI Reference Sequences ERY59288, ERZ09841.1, WP_003115311, CDG56231.1, ERX71449.1, and ERZ04935.1, respectively) optimized for expression in the host plant *Nicotiana benthamiana* were synthetized by GenScript (USA) and inserted as *Bsa*I-*Bsa*I fragments in pICH29912, assembled TMV-based magnICON^®^ vector [11] (Fig 1A). Obtained plasmids were used to transform *A*. *tumefaciens* GV3101.

### Pyocin expression in plants

*N*. *benthamiana* plants were grown in a growth chamber at 25°C and 50% humidity, with a 16 h light (1500 lux) and 8 h dark photoperiod. Four-to-six-week-old plants were used for spraying with recombinant *A*. *tumefaciens*.

*A*. *tumefaciens* were grown overnight at 30°C in LB medium containing 50 mg L^-1^ rifampicin and 50 mg L^-1^ kanamycin. *Agrobacterium* overnight cultures were adjusted to an OD_595_ of 1.5, sedimented at 3220 *g* for 5 min and resuspended in an equal volume of tap water.

Four-to-six-week-old plant leaves were sprayed with a 1:1000 dilution of *A*. *tumefaciens* strain containing expression vectors as described in Hahn et al. [12]. Spraying was performed with bacteria diluted in tap water containing 0.05% (v/v) Silwet L77 (Kurt Obermeier). Plant leaves were observed and collected at 5–10 dps.

### Purification of plant-produced pyocins

Crude protein extract was prepared as follows. A small portion of frozen leaf tissue was ground into fine powder with mortar and pestle using liquid nitrogen. Prepared powder was mixed with cold extraction buffer at a ratio of 1 g of plant material to 5 mL of buffer. The suspension was kept on ice for 15–20 min. Cell debris were removed by centrifugation at 3220 *g* at 4°C for 20 min. and the supernatant was filtered through membrane filters (pore sizes 5 μm and 0.22 μm). Obtained solution was taken as total soluble protein and applied for purification by two-step chromatography. Detailed protocols of purification are presented in Supplementary information (S1 Text). Concentration of purified proteins was evaluated by Bradford assay or by comparison of band intensity with known BSA amount run on the same SDS-PAGE gel.

### Pyocins antimicrobial activity evaluation in liquid culture

Overnight *P*. *aeruginosa* cultures were diluted to OD_595_ = 0.3 in iron-deficient Casamino Acids (CAA) medium (BD Bioscience) up to 1.2 mL. Lyophilized purified pyocins were resuspended in CAA medium, added to diluted bacterial suspension and incubated for 5.5–6.5 hours at 37°C with shaking (200 rpm). The antimicrobial activity of pyocins was evaluated by determining cell numbers of bacterial test culture. Serial dilutions of 10, 10^−1^, 10^−2^, 10^−3^, 10^−4^ and 10^−5^ were made, plated on LB agar plates, incubated overnight at 37°C and the CFU calculated.

### Soft-agar overlay assay

Overnight *P*. *aeruginosa* cultures were equalized to OD_595_ = 1.0 in CAA medium and diluted 100x in 0.8% top agar preheated in a 55°C water bath. CAA medium and Bacto Agar with low iron content (BD Bioscience) were used. Mixed overlay components were poured on plates containing solid agar (1.5% CAA agar); the plates were kept for a few minutes allowing the agar to harden. Sterile Whatman discs (6 mm diameter) were placed on soft-agar and respective amounts of pyocins (20 μl of crude extracts or 10 μg of purified pyocins) were applied to the disks. The plates were incubated overnight at 37°C and the diameter of pyocin inhibition zones were observed.

### Biofilm assays

Biofilms were grown as described by Moskowitz et al. [13], with some modifications. Briefly, *P*. *aeruginosa* strains were grown overnight in LB and diluted to OD 0.08 with fresh CAA medium. 10 μl of bacterial culture were transferred to the wells of a 96-well microtiter plate (catalog no. 269787; Nalgene Nunc International, Rochester, N.Y.) with 90 μl of CAA medium. Bacterial biofilms were formed by immersing the pegs of a modified polystyrene microtiter lid (catalog no. 445497; Nunc TSP system) into biofilm growth plates, followed by incubation controlled at 37°C for 20 h. For the treatment with pyocins, peg lids were rinsed three times in sterile water, placed into microtiter plates containing pyocins diluted in 100 μl CAA per well, and incubated for 6 h at 37°C. After incubation with pyocins, peg lids were again rinsed three times in sterile water, placed into CAA in a sterile microtiter plate and centrifuged at 810 *g* for 30 min. 6 identically treated wells were pooled each time, serial dilutions made and bacteria plated on LB plates for CFU enumeration.

To assess the adequacy of biofilm growth, the approach described by Moskovitz et al. was applied. Briefly, the biofilms grown on pegs in the biofilm growth plates were rinsed three times with water, placed in a 0.1% (wt/vol) crystal violet solution for 15 min, rinsed again, and dried for several hours. To solubilize adsorbed crystal violet, pegs with stained biofilms were incubated in 95% ethanol (150 μl per well of a flat-bottom microtiter plate) for 15 min. The absorbance was read at 590 nm on a plate reader.

### Infection of *Galleria mellonella* larvae

Overnight *P*. *aeruginosa* strains were grown in CAA medium and diluted in 0.8% NaCl in order to achieve a concentration of ~5 x 10^4^ CFU mL^-1^. Volumes of 10 μL of *P*. *aeruginosa* culture (~500 CFU) and 10 μL of pyocins solution were injected into the hemocoel of fifth instar *G*. *mellonella* larvae (Livefood UK) in proximity of the left and/or right prolegs. Pyocins were injected three hours post infection with *P*. *aeruginosa*. Injected larvae were incubated at 37°C in 9 cm Petri dishes without food for up to 5 days. Caterpillars were considered dead when they displayed no movement in response to mechanical stimulus to the head, leading to distinct changes in color from cream to dark brown/black. Twenty larvae were used per each treatment point.

## Results and discussion

Recently, Schulz et al. [10] of Nomad Bioscience (Halle, Germany) expressed in plants and characterized 12 known colicins and found that they provided excellent control of *E*. *coli* contamination in food. In response to the company's regulatory submissions, FDA recently granted plant-produced colicins a GRAS (Generally Recognized As Safe) status (GRN 593 Colicin antimicrobials for application to fruits and vegetables [https://www.accessdata.fda.gov/scripts/fdcc/index.cfm?set=GRASNotices&id=593] and GRN 676 Colicin antimicrobials for application to whole cuts and ground meat products [https://www.accessdata.fda.gov/scripts/fdcc/?set=grasnotices&id=676]), thus paving the way to rapid commercialization of colicins as a new food safety intervention for controlling foodborne *E*. *coli* infections. Because of the unmet need for new natural non-antibiotic antibacterials for controlling pathogenic *Pseudomonas aeruginosa*, we conducted a similar investigation of the colicin analogues pyocins, which are produced by members of the genus *Pseudomonas*. We expressed in plants several already described pyocins and one new pyocin, PaeM4, and studied their activity *in vitro* against planktonic and biofilm-grown bacteria and in the animal model *Galleria mellonella* (wax moth) larvae.

### The PaeM4 sequence analysis

**Fig 2:**
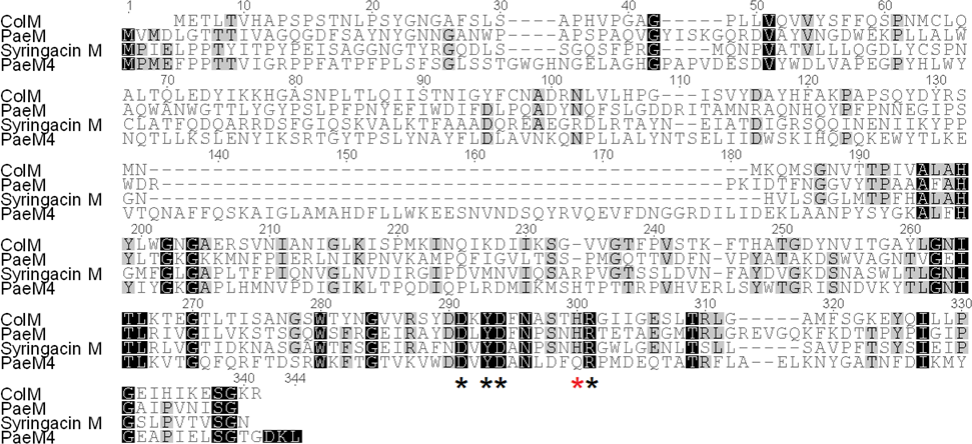
Clustal W alignment of *E*. *coli* ColM, *P*. *aeruginosa* PaeM, syringacin M and PaeM4 amino acid sequences. Residues essential for activity of ColM [19] are marked by asterisks; residues essential for activity of PaeM [20] are marked by black asterisks.

Colicin M-like bacteriocins inhibit peptidoglycan biosynthesis by degradation of lipid intermediates involved in this pathway. Colicin M-like bacteriocins from *P*. *aeruginosa* (PaeM), *P*. *syringae* (SyrM), *Burkholderia cepacia* (BurM1 and BurM2) and *Pectobacterium carotovorum* have been described [14–18]. Another colicin M distantly related gene, pyocin M4, has been identified by Ghequire & De Mot [4], but to our knowledge it has not been studied so far. This gene is found in a limited number of *P*. *aeruginosa* strains (BL01, BL03, BWPSA008, and JD332). PaeM4 sequence alignment with PaeM [14], shows only 20% amino acid identity between the two proteins (Fig 2).

Site-directed mutagenesis studies of colicin M identified residues Asp-226, Tyr-228, Asp-229, His-235, and Arg-236 as extremely important for both *in vitro* catalytic activity and *in vivo* potency [19]. All these residues are conserved also in PaeM; however, it was shown that for PaeM, mutation of His-235 affects pyocin activity only slightly [20]. In PaeM4, His-300 (analogous of His 235) is changed to Gln, but all remaining essential residues are conserved.

### Expression of pyocins in plants and purification

For pyocin expression in plants, we chose pICH29912, the assembled magnICON® TMV-based transient expression vector (Fig 1A). Extracts of *N*. *benthamiana* leaves transfected with *A*. *tumefaciens* harbouring Pyocin S5, PaeM and PaeM4 constructs and harvested at 5–7 dps (days post spraying) showed distinct bands of expected molecular weight in SDS-PAGE (56 kDa, 32 kDa and 39 kDa, respectively) (Fig 1B, left panel). All three pyocins were expressed in *N*. *benthamiana* at very high levels, comprising between 30% and 50% of total soluble leaf protein. The expression of L1, L2 and L3, the three lectin-like pyocins was further attempted and was also successful, although giving lower expression levels of 10% to 30% of total soluble leaf protein. Detected supplementary SDS-PAGE bands corresponded to expected molecular weights: 28.4 kDa for L1, 28.4 kDa for L2 and 30.3 kDa for L3. Among the three lectin-like pyocins, L3 was expressed most efficiently. Although L1 and L2 have a high degree of identity (85%), the expression level of L1 was significantly superior to the expression level of L2 (Fig 1B, right panel).

All six pyocins were purified by two-step chromatography. We obtained nearly pure pyocins with the following yields: 400 μg per gram of fresh leaf for pyocin S5, 750 μg g^-1^ for PaeM, 800 μg g^-1^ for PaeM4, 200 μg g^-1^ for L1, 60 μg g^-1^ for L2 and 400 μg g^-1^ for L3 (Fig 1C). The 55-kDa band that is seen after purification of L2 seems not to be product-related, as it can be eliminated by using a modified purification protocol: cation exchange chromatography instead of anion exchange chromatography as a second purification step. However, such purification method was not employed as it had a negative impact on the final yield of purified protein.

Thus, the non-antibiotic antibacterial proteins pyocins can be expressed at high levels in the plant *Nicotiana benthamiana*, the standard manufacturing host for multiple biopharmaceuticals currently undergoing clinical trials. The plant-produced pyocins are soluble, contains no tags, and can be easily purified by two-step protein chromatography. We believe that the plant-based process we use can be a competitive alternative to pyocin production by *E*. *coli* fermentation in terms of manufacturing economics and scalability. The expression levels reached in this study were up to 50% of the plant leaves' total soluble protein without process optimization, meaning that pyocins are not toxic to plants. In future studies, industrial procedures to enhance yield, including optimizing transfection or inducing expression in transgenic hosts, could be adopted to further increase process efficiency and lower the cost of pyocin manufacturing. These efforts have yet to be conducted. In contrast, attempts to express bacteriocin proteins in bacterial hosts have sometimes met with general toxicity challenges, even in when using expression hosts other the homologous species natively producing the bacteriocins [21,22]. This problem is most pronounced with bacteriocins with DNAse and RNAse activity, but even bacteriocins that do not express well in bacterial hosts could be successfully expressed in plants by introducing introns in the toxic domains of these proteins [10]. Thus, plants are excellent hosts for manufacturing not only phage endolysins [23–25] and colicins [10] but also pyocins. Manufacturing based on transient expression in plants has already been brought to GMP compliance, and several biopharmaceuticals produced transiently in plants are currently undergoing clinical studies, such as anti-Ebola therapeutic antibodies [26] (Prevail trial, 2016) and vaccines against non-Hodgkin’s lymphoma [27,28].

### Activity of plant-produced pyocins in agar drop plate assays

**Fig 3:**
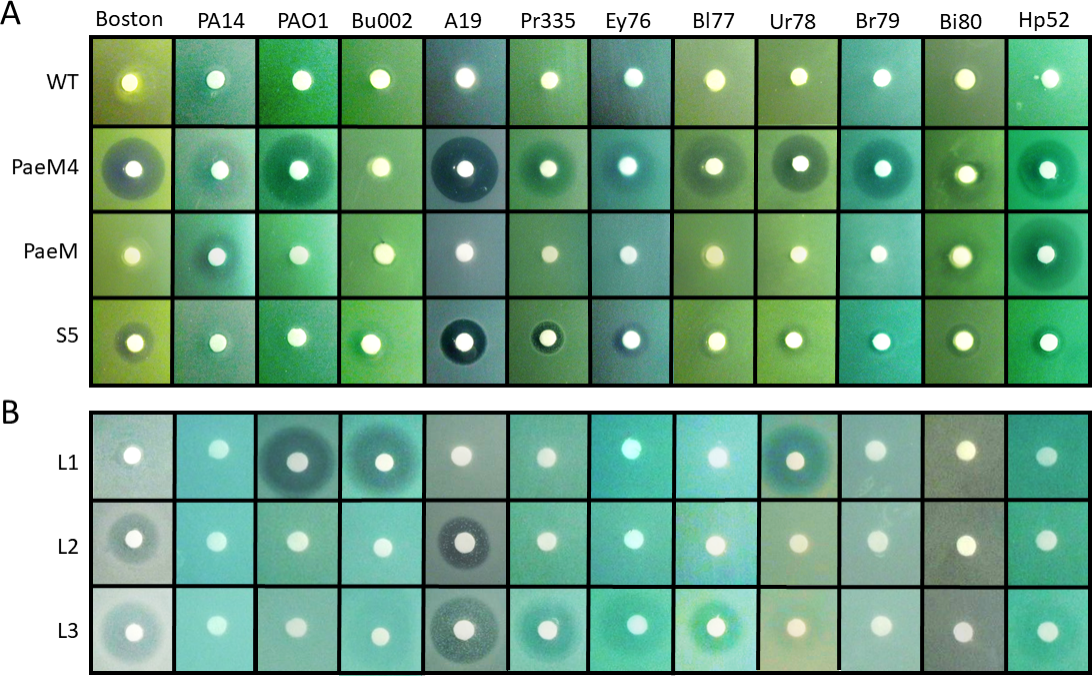
Agar drop plate assay on different *P*. *aeruginosa* isolates with plant-produced pyocins. Aliquots of 10 μl (10 μg) of purified pyocins were spotted on *P*. *aeruginosa* agar lawn and incubated overnight. **A–***P*. *aeruginosa* agar lawn grown on CAA medium, **B–***P*. *aeruginosa* agar lawn grown on LB medium.

Purified pyocins were tested at first in the agar drop plate assay with 12 *P*. *aeruginosa* strains. We used iron-deficient CAA medium for S5, PaeM and PaeM4 pyocins, which are known (S5 and PaeM) or expected (PaeM4) to use the iron receptor. For lectin-like pyocins, LB medium was used. All six plant-expressed pyocins formed inhibition zones on several *P*. *aeruginosa* strains. Inhibition zones were of different width and haziness, depending on the strain and the pyocin. PaeM4 formed large inhibition zones on almost all tested strains. Pyocins S5 and L3 also demonstrated wide ranges of activity, while pyocins PaeM, L1 and L2 had narrower specificity spectra (Fig 3A and 3B).

**Fig 4:**
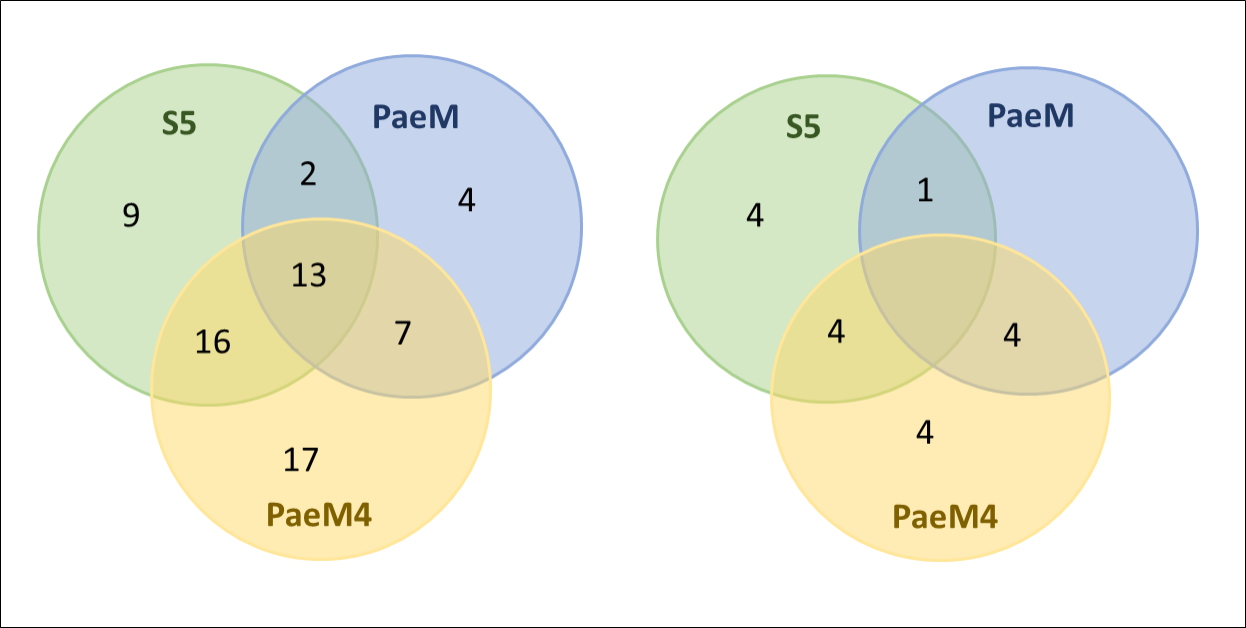
Sensitivity of clinical isolates to three plant-expressed pyocins. Venn diagram describing pyocin susceptibilities of clinical *Pseudomonas aeruginosa* isolates. Left–susceptibility of all 100 tested isolates, right–susceptibility of 21 antibiotic-resistant isolates.

To confirm initial results that showed that PaeM4 has significantly wider specificity than PaeM, and that the above-mentioned results were not biased by our initial choice of strains, we conducted a larger activity study. In this secondary inhibition screen, 100 clinical isolates of *P*. *aeruginosa* were tested in the agar drop plate assay using purified plant-made pyocins. Pyocin S5 formed inhibition zones on 40% of strains tested, while PaeM inhibited 26% of the strains. PaeM4 inhibited 53% of the strains, demonstrating again the widest range of control among the three bacteriocins. By using only three pyocins we could target up to 68% of all tested clinical isolates of *P*. *aeruginosa* (Fig 4 and S1 Table).

We then looked more closely at the antibiotic resistance spectrum of the clinical isolates to ascertain whether pyocins were equally active against antibiotic susceptible and resistant strains. Twenty-one isolates out of the 100 tested were known to be resistant to at least three antibiotics. It appeared that 43% (9 of 21) of the resistant strains were targeted by pyocin S5, 24% (5 of 21) were sensitive to PaeM, and 58% (12 of 21) were targeted by PaeM4 (Figs 4 and S1). We then looked at pyocin sensitivity of carbapenem-resistant strains. Twenty-one strains in the tested panel were carbapenem resistant, and 14 of these strains (67%) were sensitive to at least one of the three tested pyocins (S1 Table). These numbers are very close to the sensitivity of all 100 of the strains tested in general and suggest that antibiotic resistance in *P*. *aeruginosa* strains does not influence their sensitivity to pyocins.

Thus, plant-expressed pyocins, like the earlier reported plant-produced *E*. *coli*-analogues colicins, are fully functional antibacterials. Furthermore, our results allow us to envisage the use of pyocins to treat infections caused by carbapenem-resistant and even multi-drug resistant *P*. *aeruginosa* serotypes. We show that simple mixture of three pyocins, applied at low concentrations, are highly and broadly active against 68 of the 100 pathogenic *P*. *aeruginosa* isolates tested. These studies can be extended in the future to include additional pyocins, which collectively could allow for even higher pathogen coverage.

### PaeM4 and PaeM_NCTC10332_ use different receptors

It has been recently demonstrated that ColM-like *Pseudomonas* bacteriocins PaeM (NCTC10332) and PmnH target ferrichrome receptor FiuA [29]. PaeM4 demonstrates significantly higher activity when *P*. *aeruginosa* is grown in medium with reduced iron content, thus suggesting the involvement of some siderophore receptor.

**Fig 5:**
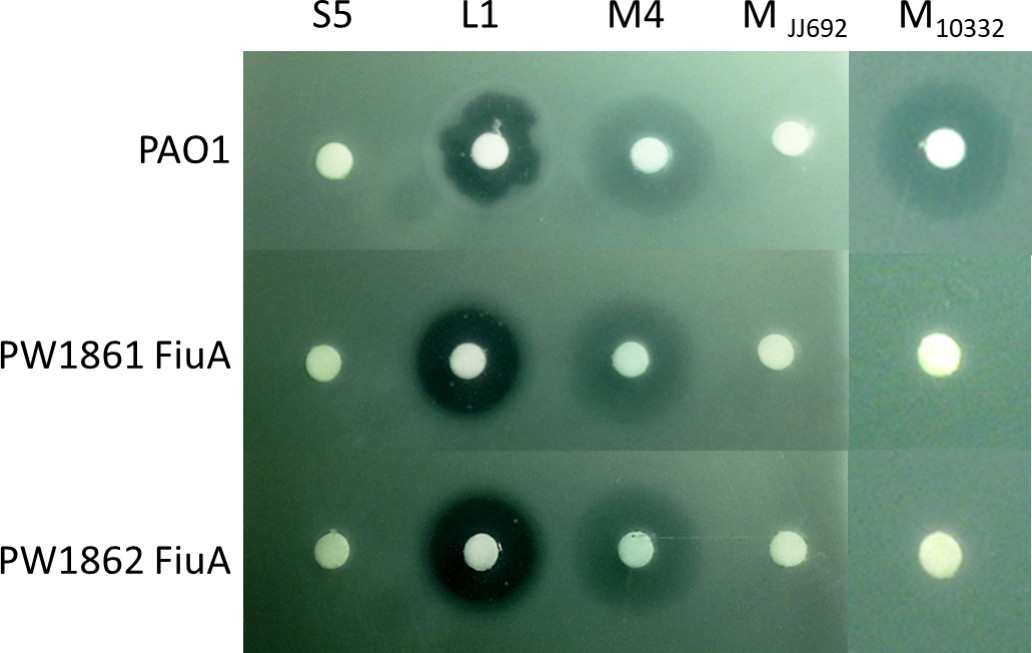
Sensitivity of PAO1 and PAO1 (FiuA) to pyocins. Plant-produced pyocins S5, L1, PaeM4, PaeM_JJ692_ and PaeM _NCTC10332_ were applied (5 μg each) on *P*. *aeruginosa* PAO1, PW1861 (PAO1 (FiuA)) and PW1862 (PAO1 (FiuA)) lawn and the plates were incubated overnight at 37°C.

To verify whether FiuA is involved in the PaeM4 entry mechanism, we tested PAO1 transposon insertion FiuA mutants PW1861 and PW1862 for sensitivity to PaeM4 [30,31]. PaeM4 forms a hazy inhibition zone on a lawn of wild type PAO1; if FiuA is indeed the PaeM4 receptor, this zone should be absent on transposition mutants. However, the PaeM4 inhibition zone was equally detected also on FiuA mutants (Fig 5). Thus, PaeM4 must be using a different receptor than FiuA.

Interestingly, when we tested activity of PaeM_JJ692_ in the same assay we realized that PaeM from *P*. *aeruginosa* strain JJ692 did not form inhibition zone not only of FiuA mutants, but also on wild type PAO1 lawn. We then tested PaeM from *P*. *aeruginosa* strain NCTC10332, which has been used in similar study by Ghequire et al. [29]. As expected, PaeM_NCTC10332_ formed inhibition zone on wild type PAO1 lawn but not on FiuA mutants PW1861 and PW1862, demonstrating involvement of FiuA in reception of this pyocin. It appears that PaeM_NCTC10332_ and PaeM_JJ692_ have different specificity despite their high sequence homology (90% of a.a. identity). Additional studies are needed to explain the reason of this different specificity (involvement of different receptors, or immunity proteins). From practical point of view, these results suggest that expressing a panel of homologous pyocins from different *P*. *aeruginosa* strains might significantly enlarge the breath and potency of antimicrobial product we are developing.

### Pyocin susceptibility assays in liquid nutrient medium

We next evaluated the susceptibility of *P*. *aeruginosa* strains to plant-expressed pyocins in liquid culture assays. For this experiment, we selected three *P*. *aeruginosa* strains demonstrating different pyocin sensitivities. The A19 strain was chosen because in agar drop plate assays it demonstrated sensitivity to the highest number of pyocins (S5, PaeM4, L2 and L3). PAO1 strain is a producer of pyocin S5 and is immune to this pyocin; it cannot be targeted by PaeM but is sensitive to PaeM4 and L1. The hospital pneumoniae isolate HP52 is sensitive only to *P*. *aeruginosa* colicin M homologs, PaeM and PaeM4. Before starting the experiment, we determined the minimum inhibitory concentration (MIC) of pyocins S5 and PaeM4 for the A19 strain in order to approximate the treatment concentration. An MIC of 0.1 μg mL^-1^ was determined for pyocin S5 and 0.6 μg mL^-1^ for PaeM4 (S1 Fig). Thus, we used 5 μg mL^-1^ as the treatment dose, which is 5 to 10-fold the determined MIC.

**Fig 6:**
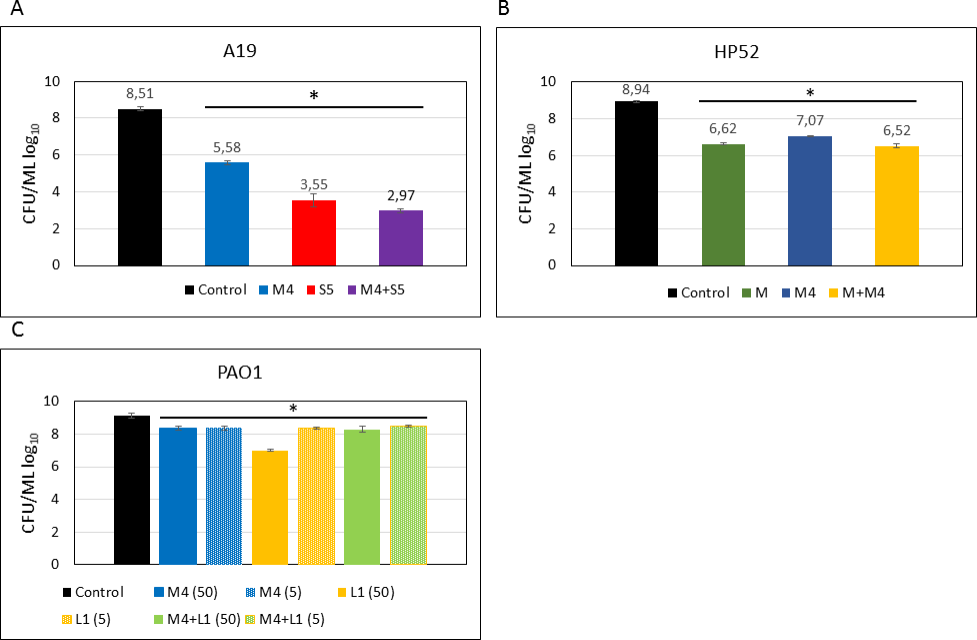
Pyocin antibacterial assays in liquid culture. *P*. *aeruginosa* strains were cultivated in CAA medium, treated with 5 μg mL^-1^ or 50 μg mL^-1^ of one or several pyocins and incubated with shaking for 6.5 hours. The antimicrobial activity of pyocins was evaluated by determining cell numbers of bacterial test culture. Serial dilutions of 10, 10^−1^, 10^−2^, 10^−3^, 10^−4^ and 10^−5^ were made, plated on LB agar plates, incubated overnight at 37°C and the CFU calculated. Data are the mean ± SD of three independent experiments. * Denotes statistical significance (p≤0.001) for comparison of treatment with antimicrobials versus control by a one-way ANOVA test with Bonferroni correction applied.

In this experiment, the A19 strain was found highly susceptible to PaeM4 and S5. Five μg mL^-1^ of pyocins added to CAA medium reduced bacterial CFU counts by several orders of magnitude: PaeM4 treatment achieved CFU reduction of almost three orders of magnitude, S5 treatment achieved CFU reduction of five orders of magnitude, and a mixture of both pyocins reduced the CFU count by as much as 5.5 log_10_ (Fig 6A). PaeM4 with Gln-300 mutated to His-300 was also used in parallel in this assay to verify whether this mutation could improve the potency of PaeM4. However, no significant difference in potency was found between the mutated and native forms of PaeM4 in the liquid culture assays (S2 Fig). In contrast to the high efficacy *in vitro* shown by PaeM4 and S5, treatment of A19 with either of the lectin-like pyocins L2 and L3 demonstrated only slight reductions in CFU of 0.3–0.4 log_10_ (S3 Fig).

Treatment of strain HP52 with PaeM and PaeM4 yielded similar results for both pyocins, with slightly better efficacy *in vitro* for PaeM. Addition of 5 μg mL^-1^ of PaeM reduced bacterial CFU by about two orders of magnitude (Fig 6B). The CFU reduction achieved by a mixture of both pyocins had no cumulative effect and was similar to that obtained by either pyocins alone (2.4 log_10_).

*P*. *aeruginosa* PAO1 strain is sensitive to L1 and PaeM4, with L1 forming clear inhibition zones and PaeM4 forming hazy inhibition zones in the agar drop test assay. The sensitivity assay in liquid PAO1 culture demonstrated less than 1 log_10_ CFU reduction after treatment with 5 or 50 μg mL^-1^ of PaeM4. L1 treatment was more effective, providing >2-log CFU reduction when 50 μg mL^-1^ pyocin was used for the treatment. Exposure of bacteria to a mixture of both pyocins yielded similar results as PaeM4 treatment alone (Fig 6C).

### Activity of pyocins against *P*. *aeruginosa* biofilms

The emergence of pan-resistant strains of Gram-negative bacteria and the ability of many pathogens to form multidrug-resistant biofilms during infection increases the threat of bacterial diseases that are untreatable with current antibiotics [32]. Biofilm formation is the most common growth mode adopted by many bacteria and is now recognized to be of high clinical significance [33]. A biofilm is a matrix-enclosed bacterial population in which bacteria adhere both to each other and also to surfaces or interfaces [34,35]. Prominent chronic diseases in which bacterial biofilm formation enables persistence within the host and contributes to pathogenesis include, among others, *Pseudomonas aeruginosa* lung infection in patients with cystic fibrosis (CF) [36,37]. Smith et al. [6] were the first to show that pyocin S2 displays potent activity against *P*. *aeruginosa* biofilms, suggesting a potentially improved therapeutic option.

**Fig 7:**
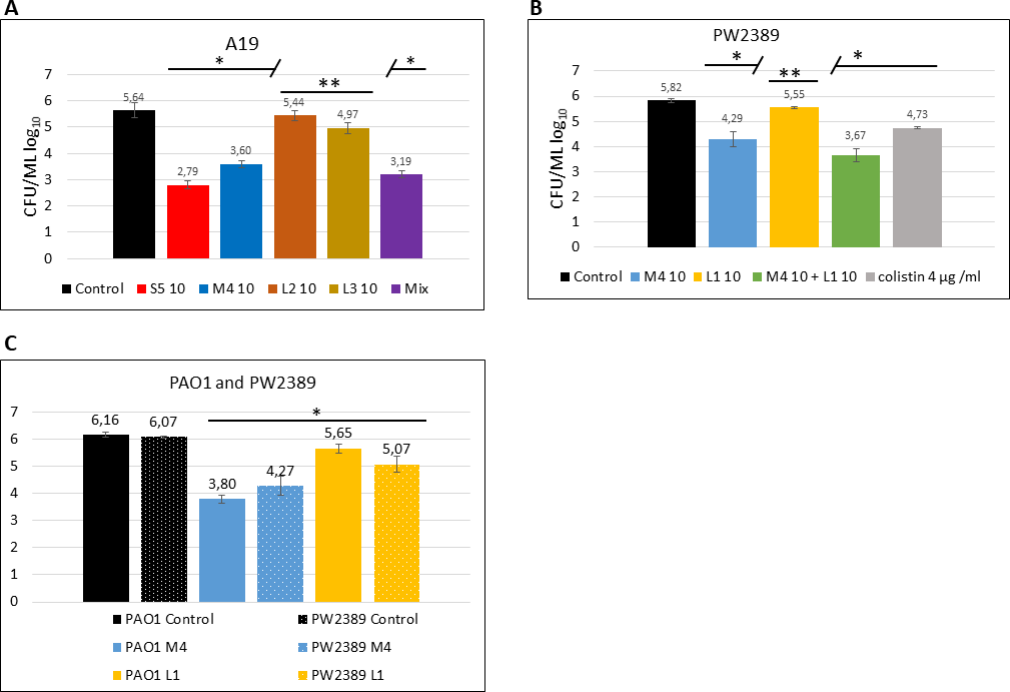
Pyocin activity against biofilms. One day-old *P*. *aeruginosa* biofilms grown in CAA medium were treated with pyocins. **A–**A19 strain treated with 10 μg mL^-1^ of pyocins, when each pyocin is used separately, and 2.5 μg of each pyocin used as a mixture. **B**–PW2389 (PAO1(mucB)) treated with 100 μg mL^-1^ of pyocins. **C**–PAO1 and PW2389 treated with 10 μg mL^-1^ of pyocins. Data are the mean ± SD of at least three separate independent experiments. * Denotes statistical significance (p≤0.001) for comparison of treatment with antimicrobials versus control by a one-way ANOVA test with Bonferroni correction applied. ** no statistical significance (p>0.001).

We examined the activity of pyocins in young, one day old *P*. *aeruginosa* biofilms grown under iron-limited conditions. At first, the ability of *P*. *aeruginosa* strains to form biofilms in CAA medium was evaluated by crystal violet staining (S4 Fig). After confirming that A19 and PAO1 strains both formed biofilms, these strains were used in the biofilm eradication assessment. Biofilms of strain A19 were exposed to pyocins S5, M4, L2 and L3, and to combination of all four pyocins. Pyocin S5 was the most powerful in eradicating biofilm and reduced the CFU count by almost three orders of magnitude. Pyocin PaeM4 reduced biofilm CFU count by two orders of magnitude. As in liquid culture experiments, both lectin-like pyocins L2 and L3 did not demonstrated significant activity in reducing CFU counts. Equal doses of the four pyocins at 25% w/w (2.5 μg mL^-1^) each administered as a mixture (10 μg mL^-1^ total) was almost as efficient in eradicating biofilm as 10 μg mL^-1^ of pyocin S5 (Fig 7A).

Most cystic fibrosis isolates growing as biofilms in human lung are mucoid strains and produce alginate. *P*. *aeruginosa* mucB mutants have mucoid phenotype and produce alginate when grown in iron-reduced conditions [38]. To assess how mucoidy may influence sensitivity to pyocins, we used PAO1 mucB transposition mutant strain PW2389 [30,31] in biofilm experiments. In young biofilms, treatment with 100 μg mL^-1^ of PaeM4 and PaeM4 combined with L1 reduced PW2389 biofilm CFU count by 1.5–2 log_10_. Treatment with L1 alone did not change CFU counts significantly (Fig 7B).

We repeated the experiment with PAO1 and PAO1 (mucB) in parallel with lower concentrations of pyocins. As in the previous experiment, despite a ten-fold lower concentration, PaeM4 treatment reduced CFU counts with very similar efficiency. This reduction was slightly more pronounced for the non-mucoid PAO1 strain. L1 had little effect on the CFU counts of either strains (Fig 7C). In conclusion, under the experimental conditions used, the mucoidy of *P*. *aeruginosa* had no significant effect on pyocins' activity in biofilms.

More detailed analyses with biofilms are necessary, including screening different *P*. *aeruginosa* strains as well as effects on mature biofilms. However, these preliminary results clearly indicate that plant-expressed pyocins and pyocins in general could be used as antibacterials to eradicate *P*. *aeruginosa* biofilms.

### Pyocin activity in *Galleria mellonella* challenge assays

*Galleria melonella* larvae have already been used as a *P*. *aeruginosa* challenge model. Using this invertebrate model of *P*. *aeruginosa* infection, Smith et al. [6] demonstrated that pyocin S2 is highly active *in vivo* and is capable of protecting against a lethal *P*. *aeruginosa* infection.

**Fig 8:**
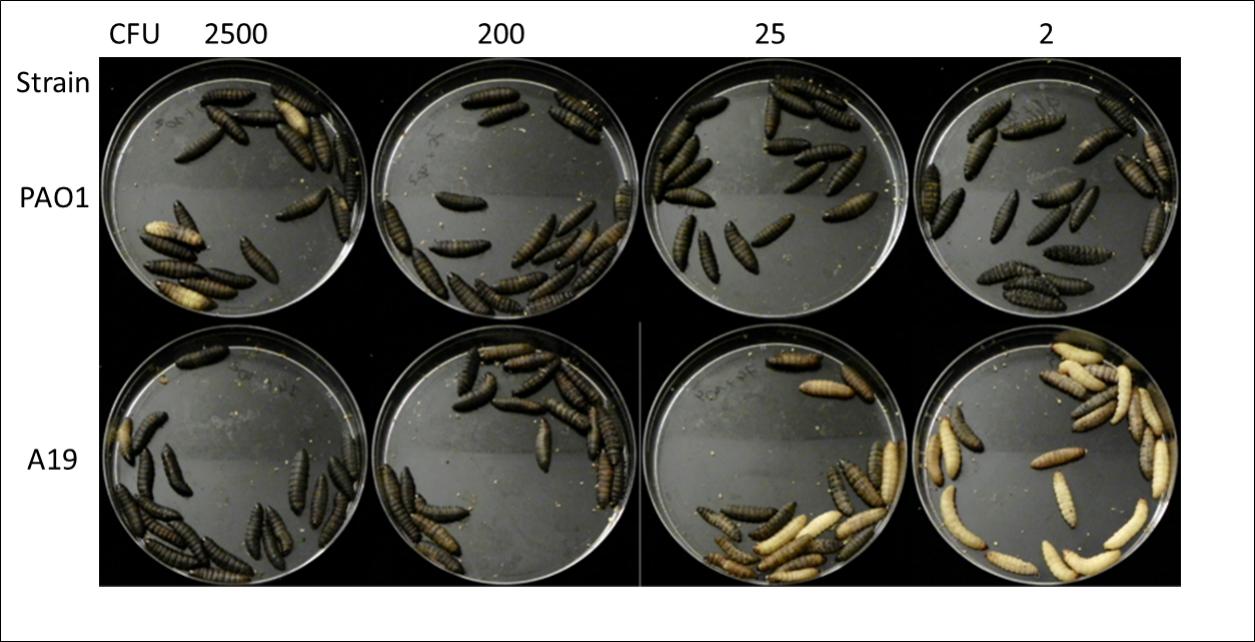
Survival of *Galleria melonella* larvae after injection with *P*. *aeruginosa* PAO1 or A19. Fifth-instar *G*. *mellonella* larvae were injected in the left hind proleg with different amounts of *P*. *aeruginosa* PAO1 and A19 bacteria. The image was captured 18 hours post infection. Healthy larvae are cream colored, and darker pigmentation indicates infection. Dead larvae can be recognized from their dark brown-black color.

Prior to administration of pyocins, we sought to determine the minimal lethal dose for the larvae of *P*. *aeruginosa* strains PAO1 and A19 (Fig 8). It appears that as little as ~2 CFU of *P*. *aeruginosa* PAO1 are sufficient to kill all the larvae in 18 hours (LD_100_ = ~2 CFU). Strain A19 is less virulent, with ~200 CFU needed to achieve complete killing of the insects after 18 hours (LD_100_ = ~200 CFU). However, when incubation was extended to 24 hours, all larvae were dead also after infection with ~2 CFU of A19, demonstrating the high sensitivity of *Galleria mellonella* to *P*. *aeruginosa* infection in general.

**Fig 9:**
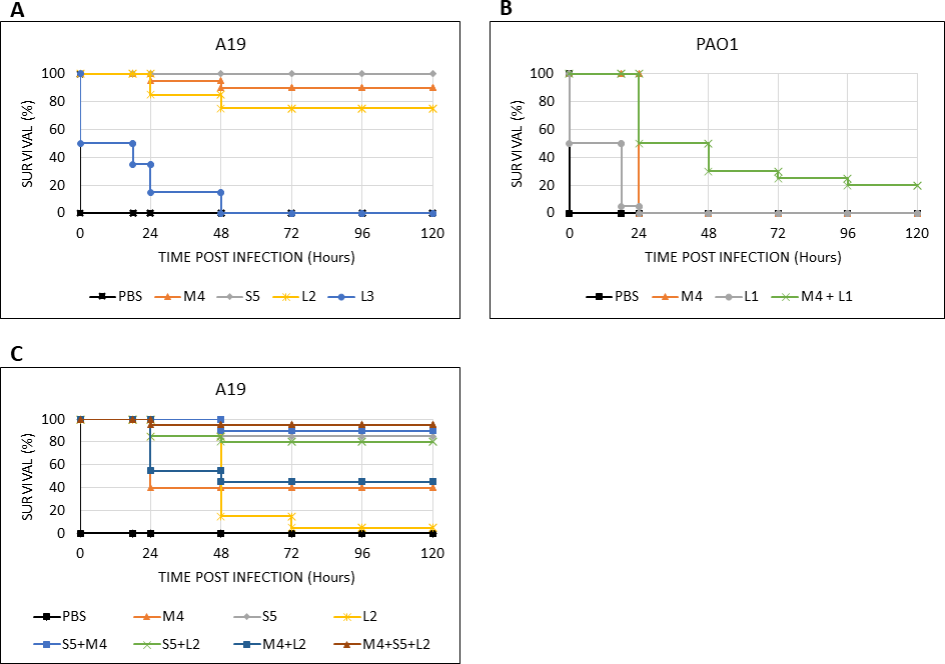
Survival of larvae pre-infected with *P*. *aeruginosa* PAO1 and A19 and treated therapeutically with pyocins. *G*. *melonella* larvae were infected with 500 CFU of *P*. *aeruginosa* A19 or PAO1 and treated with pyocins 3 hours after infection. **A–**larvae infected with A19 strain and treated with 10 μg of the indicated pyocin. **B–**larvae infected with PAO1 strain and treated with 10 μg of the indicated pyocin. **C–**larvae infected with A19 strain and treated with 1 μg of the indicated pyocin. Killing curves were plotted and estimation of differences in survival analyzed by the Kaplan-Meier method using XLSTAT software.

Next, we tested the ability of PaeM4 to rescue larvae from infection with *P*. *aeruginosa* PAO1 and A19 strains (Fig 9). 500 CFU of each strain were used separately for infection, which corresponds to about 250-times the LD_100_ (18 h) for PAO1 and 2.5-times the LD_100_ (18 h) for A19. Pyocins were injected three hours after infection with *P*. *aeruginosa*. Larvae infected with the A19 strain were treated with 10 μg each of pyocins S5, PaeM4, L2 and L3. All larvae in the control group not treated by pyocins were dead in 18 hours, as expected. Pyocin S5 treatment rescued all larvae from infection (100% efficacy). PaeM4 treatment rescued 90% of larvae and L2 treatment rescued 75% of larvae. Treatment with pyocin L3 prolonged the survival of the larvae significantly (all dead at 96 h vs 18 h for the control larvae), but the treatment did not rescue the animals as did treatment with the other pyocins (Fig 9A). The results obtained with L2 are somehow surprising because this pyocin was only slightly active in reducing CFU counts in liquid medium and in biofilm experiments. The relatively high activity of this pyocin in the insect model might signify that additional unknown factors may impact the activity of this lectin. These results suggest that the animal model might reflect pharmaceutical activities of pyocins more adequately than *in vitro* experiments.

Pyocins PaeM4 and L1 were used in PAO1 larval challenge experiments. In this experiment, neither of two pyocins were able to rescue larvae from infection completely. However, both pyocins significantly extended the survival of the larvae. PaeM4 completely protected all larvae for 24 hours post infection, but all larvae succumbed to infection after 48 hours. L1 protected 50% of larvae for 18 hours, but almost all animals were dead after 24 hours. However, when both pyocins PaeM4 and L1 were used in a mixture, the survival was significantly improved in comparison with either pyocin applied alone: 50% of larvae survived 48 hours post infection and 20% of larvae survived until the end of experiment (Day 5) (Fig 9B).

We repeated the larval challenge experiments with tenfold lower doses of pyocins and strain A19. After treatment with 1 μg PaeM4, 40% of larvae survived. At the same low dose, pyocin S5 alone rescued 85% of the larvae, while pyocin L2 did not rescue the larvae but did prolong their survival. Cocktails of pyocins did lead to improved survival, with 95% of larvae protected and alive at 120 hours post exposure when all three pyocins were used (Fig 9C).

In general, the results obtained are very encouraging. The difference in survival rates between the two tested strains of *P*. *aeruginosa*, PAO1 and A19, reflects the different virulence of these strains. The extreme sensitivity of wax moth larvae to *P*. *aeruginosa* requires complete killing of all bacteria, which is hardly achievable in real life. However, the *Galleria mellonella* model provides an excellent tool for pre-screening antimicrobial candidates and should help reduce the number of experiments with mammalian species. Relative to screening inhibitory activity *in vitro*, the larval model may be more closely aligned with the mouse model for *P*. *aeruginosa* infection and control, which in turn might be more relevant to how humans may be treated with pyocins in a clinical setting.

## Conclusion

*Pseudomonas aeruginosa* is among the leading causes of mortality from nosocomial infections in the United States and worldwide. We investigated pyocins, natural non-antibiotic antimicrobial proteins produced by certain *P*. *aeruginosa* strains and active against other strains of the species, as potential pathogen control agents. We demonstrate that plant-expressed pyocins, like their earlier reported *E*. *coli*-produced analogues colicins, can be expressed at high yields in plants and are fully functional. We show that a simple mixture of three pyocins is broadly active against as much as 68% of the tested pathogenic *P*. *aeruginosa* isolates. The breadth of activity and potency of pyocins might be further improved by engineering the proteins by swapping their receptor and activity domains. For example, Pyocin S5, which demonstrates high antimicrobial activity, might be made potent against a greater number of strains by exchanging its receptor binding domain by receptor binding domains of other pyocins (for example, S2 or S3) [39]. Importantly, pyocins are effective against biofilm-forming bacteria.

In situations when the microbiology of the infection is monitored and the *P*. *aeruginosa* serotype is identified, strain-specific protein antibacterials such as pyocins may provide a useful therapeutic option; in some cases, such an option might be life-saving. Our data confirm and extend these observations with new pyocins produced efficiently in plants, and evaluated singly and as mixtures against pathogen strains grown both planktonically and as biofilms. We propose that plant-produced pyocins should be considered as a viable alternative to antibiotics for the control of pathogenic *P*. *aeruginosa*.

## Supporting information

S1 Table *P*. *aeruginosa strains* used in the study. The strains used for pyocins activity study (Fig 4) are listed in the shaded part of the table.
(PDF)

S1 Text Purification of plant-produced pyocins. (PDF)

S1 Fig Determination of pyocin MIC by agar dilution method against *Pseudomonas aeruginosa* strain A19. (PDF)

S2 Fig PaeM4 and PaeM4H liquid culture killing assay. (PDF)

S3 Fig L2 and L3 liquid culture killing assay. (PDF)

S4 Fig *P*. *aeruginosa* biofilms. (PDF)
