# Supplementary material for: Plant-expressed pyocins for control of Pseudomonas aeruginosa
Source: PLoS One. 2017 Oct 3;12(10):e0185782. doi: 10.1371/journal.pone.0185782 (PMC5626474; doi:10.1371/journal.pone.0185782)
Supplement: S1 Table — The strains used for pyocins activity study (Fig 4) are listed in the shaded part of the table. (PDF) [file pone.0185782.s001.pdf]

S1 table. ***P. aeruginosa strains* used in the study.**

***P. aeruginosa* strain**	**Provenance**	**Antibiotic resistance***	**S5**	**M**	**M4**	**L1**	**L2**	**L3**
Boston strain	ATCC27853, Blood culture		+	-	+	-	+	(+)
PAO1	ATCC15692, Infected wound		-	-	+	+	-	-
PA14	DSM19882		-	+	+	-	-	-
Bu002	LMG24892 Infected wound		+	-	-	+	-	-
A19	LMG25088 Infected wound		+	-	+	-	+	+
Pr335	LMG24969 Hospital environment		+	-	+	-	-	+
PW1861 (FiuA)	Manoil lab *P. aeruginosa* mutant library, University of Washington	TCN						
PW2387 (FiuA)	Manoil lab *P. aeruginosa* mutant library, University of Washington	TCN						
PW2389 (mucB)	Manoil lab *P. aeruginosa* mutant library, University of Washington	TCN						
PW2388 (mucB)	Manoil lab *P. aeruginosa* mutant library, University of Washington	TCN						
EY76	Infected eye	-	+	-	+	-	-	-
BL77	Blood	-	+	-	+	-	-	+
UR78	Urinary tract infection	-	+	-	+	+	-	-
BR79	Bronchus	-	+	-	+	-	-	-
BI80	Biopsy	-	+	+	+	-	-	-
HP1	Hospital pneumonia	-	+	-	+	n.t.	n.t.	n.t.
HP2	Hospital pneumonia	IPM, MEM, CIP	+	-	-	n.t.	n.t.	n.t.
HP3	Hospital pneumonia	-	-	-	-	n.t.	n.t.	n.t.
HP4	Hospital pneumonia	PIP, TZP, CIP, CAZ, FEP, IPM, MEM	+	-	+	n.t.	n.t.	n.t.
PU5	Pus	-	-	-	-	n.t.	n.t.	n.t.
HP6	Hospital pneumonia	-	+	-	+	n.t.	n.t.	n.t.
HP7	Hospital pneumonia	-	+	-	+	n.t.	n.t.	n.t.
UR8	Urinary tract infection	PIP, TZP, CIP	+	-	-	n.t.	n.t.	n.t.
UR9	Urinary tract infection	-	-	-	-	n.t.	n.t.	n.t.
UR10	Urinary tract infection	-	+	-	+	n.t.	n.t.	n.t.
HP11	Hospital pneumonia	MEM	-	-	-	n.t.	n.t.	n.t.
PU12	Pus	IPM, MEM, CIP, GM, NN	-	+	+	n.t.	n.t.	n.t.
PU13	Pus	-	+	+	+	n.t.	n.t.	n.t.
HP14	Hospital pneumonia	-	-	-	-	n.t.	n.t.	n.t.
HP15	Hospital pneumonia	-	-	-	-	n.t.	n.t.	n.t.
PU16	Pus	-	+	+	-	n.t.	n.t.	n.t.
HP17	Hospital pneumonia	-	-	-	-	n.t.	n.t.	n.t.
HP18	Hospital pneumonia	PIP, TZP, CIP	+	-	+	n.t.	n.t.	n.t.

HP19	Hospital pneumonia	PIP, TZP, CAZ, FEP, CIP	+	-	+	n.t.	n.t.	n.t.
HP20	Hospital pneumonia	-	-	-	-	n.t.	n.t.	n.t.
HP21	Hospital pneumonia	-	-	-	-	n.t.	n.t.	n.t.
UR22	Urinary tract infection	PIP, TZP, CIP	-	-	-	n.t.	n.t.	n.t.
HP23	Hospital pneumonia	PIP, TZP, CIP, FEP, CAZ, IPM, MEM	+	+	-	n.t.	n.t.	n.t.
UR24	Urinary tract infection	CIP	+	+	+	n.t.	n.t.	n.t.
HP25	Hospital pneumonia	-	-	-	-	n.t.	n.t.	n.t.
UR26	Urinary tract infection	-	-	-	-	n.t.	n.t.	n.t.
UR27	Urinary tract infection	-	+	+	+	n.t.	n.t.	n.t.
UR28	Urinary tract infection	IPM, MEM, CIP	+	-	+	n.t.	n.t.	n.t.
UR29	Urinary tract infection	-	-	-	-	n.t.	n.t.	n.t.
UR30	Urinary tract infection	-	-	-	-	n.t.	n.t.	n.t.
HP31	Hospital pneumonia	-	-	-	-	n.t.	n.t.	n.t.
HP32	Hospital pneumonia	-	-	-	-	n.t.	n.t.	n.t.
HP33	Hospital pneumonia	-	-	-	-	n.t.	n.t.	n.t.
PU34	Pus	PIP, TZP, CAZ, MEM, SCP	-	-	+	n.t.	n.t.	n.t.
HP35	Hospital pneumonia	-	+	-	-	n.t.	n.t.	n.t.
PU36	Pus	-	+	+	+	n.t.	n.t.	n.t.
PU37	Pus	CIP	-	-	+	n.t.	n.t.	n.t.
HP38	Hospital pneumonia	-	+	-	+	n.t.	n.t.	n.t.
HP39	Hospital pneumonia	-	+	-	-	n.t.	n.t.	n.t.
HP40	Hospital pneumonia	-	+	+	+	n.t.	n.t.	n.t.
HP41	Hospital pneumonia	-	+	+	+	n.t.	n.t.	n.t.
UR42	Urinary tract infection	-	-	-	-	n.t.	n.t.	n.t.
UR43	Urinary tract infection	-	+	+	+	n.t.	n.t.	n.t.
PU44	Pus	-	+	+	+	n.t.	n.t.	n.t.
PU45	Pus	PIP, MEM	-	-	-	n.t.	n.t.	n.t.
PU46	Pus	-	+	-	-	n.t.	n.t.	n.t.
HP47	Hospital pneumonia	-	+	+	+	n.t.	n.t.	n.t.
PU48	Pus	-	-	+	-	n.t.	n.t.	n.t.
HP49	Hospital pneumonia	PIP, TZP, CAZ	-	-	-	n.t.	n.t.	n.t.
HP50	Hospital pneumonia	MEM, IPM	-	-	+	n.t.	n.t.	n.t.
UR51	Urinary tract infection	PIP, TZP, CIP, GM, IPM, MEM, NN, SCP	-	-	-	n.t.	n.t.	n.t.
HP52	Hospital pneumonia	-	-	+	+	n.t.	n.t.	n.t.
HP53	Hospital pneumonia	-	-	-	+	n.t.	n.t.	n.t.
HP54	Hospital pneumonia	-	-	-	+	n.t.	n.t.	n.t.
HP55	Hospital pneumonia	IPM, CIP	-	-	-	n.t.	n.t.	n.t.
UR56	Urinary tract infection	GM, NN, CIP	-	-	+	n.t.	n.t.	n.t.
PU57	Pus	-	-	-	+	n.t.	n.t.	n.t.
PU58	Pus	-	-	-	+	n.t.	n.t.	n.t.
BL59	Blood	-	-	-	-	n.t.	n.t.	n.t.
HP60	Hospital pneumonia	-	+	-	+	n.t.	n.t.	n.t.

HP61	Hospital pneumonia	-	-	+	+	n.t.	n.t.	n.t.
HP62	Hospital pneumonia	-	+	-	+	n.t.	n.t.	n.t.
HP63	Hospital pneumonia	-	-	-	+	n.t.	n.t.	n.t.
HP64	Hospital pneumonia	IPM, MEM	+	-	+	n.t.	n.t.	n.t.
HP65	Hospital pneumonia	IPM, MEM, CIP	+	-	-	n.t.	n.t.	n.t.
PU66	Pus	-	+	-	+	n.t.	n.t.	n.t.
UR67	Urinary tract infection	-	-	-	-	n.t.	n.t.	n.t.
UR68	Urinary tract infection	-	+	-	-	n.t.	n.t.	n.t.
BL69	Blood	CIP, MEM	-	-	-	n.t.	n.t.	n.t.
UR70	Urinary tract infection	PIP	+	-	-	n.t.	n.t.	n.t.
HP71	Hospital pneumonia	CIP	-	-	+	n.t.	n.t.	n.t.
HP72	Hospital pneumonia	PIP, TZP, CAZ, MEM, CIP, SCP	-	-	-	n.t.	n.t.	n.t.
HP73	Hospital pneumonia	-	-	-	-	n.t.	n.t.	n.t.
HP74	Hospital pneumonia	-	-	+	-	n.t.	n.t.	n.t.
HP75	Hospital pneumonia	CAZ, FEP, SCP	-	+	+	n.t.	n.t.	n.t.
HP76	Hospital pneumonia	-	+	+	-	n.t.	n.t.	n.t.
HP77	Hospital pneumonia	PIP, TZP, CAZ, FEP, MEM, IPM, SCP	-	+	+	n.t.	n.t.	n.t.
PU78	Pus	-	+	+	+	n.t.	n.t.	n.t.
HP79	Hospital pneumonia	-	-	+	-	n.t.	n.t.	n.t.
PU80	Pus	-	-	-	+	n.t.	n.t.	n.t.
UR81	Urinary tract infection	-	-	+	+	n.t.	n.t.	n.t.
PU82	Pus	CIP	+	+	+	n.t.	n.t.	n.t.
PU83	Pus	-	+	+	+	n.t.	n.t.	n.t.
HP84	Hospital pneumonia	-	-	+	-	n.t.	n.t.	n.t.
HP85	Hospital pneumonia	-	-	-	-	n.t.	n.t.	n.t.
HP86	Hospital pneumonia	PIP, TZP, SCP, CAZ, FEP, IPM, MEM	+	-	-	n.t.	n.t.	n.t.
HP87	Urinary tract infection	IPM, MEM	-	-	+	n.t.	n.t.	n.t.
HP88	Hospital pneumonia	IPM, MEM, CIP	-	-	+	n.t.	n.t.	n.t.
UR89	Urinary tract infection	-	+	-	+	n.t.	n.t.	n.t.
PU90	Pus	-	-	+	+	n.t.	n.t.	n.t.
UR91	Urinary tract infection	CAZ, CIP, PIP, TZP	-	-	+	n.t.	n.t.	n.t.
HP92	Hospital pneumonia	IPM, MEM, CIP, NN, GM	-	+	+	n.t.	n.t.	n.t.
UR93	Urinary tract infection	-	-	-	-	n.t.	n.t.	n.t.
UR94	Urinary tract infection	-	-	-	+	n.t.	n.t.	n.t.
PU95	Pus	-	-	-	+	n.t.	n.t.	n.t.
HP96	Hospital pneumonia	-	-	-	-	n.t.	n.t.	n.t.
HP97	Hospital pneumonia	IPM, MEM	-	-	-	n.t.	n.t.	n.t.
HP98	Hospital pneumonia	-	-	-	+	n.t.	n.t.	n.t.
UR99	Urinary tract infection	-	+	+	+	n.t.	n.t.	n.t.
HP100	Hospital pneumonia	-	-	-	-	n.t.	n.t.	n.t.

*Antibiotic abreviations:

TCN (tetracycline); Carbapenems: IPM (imipenem); MEM (meropenem); Fluoroquinolones: CIP (ciprofloksacin); Penicillin/Ureidopenicillin: PIP (piperacilin); β-lactam/β-lactamase inhibitor combination: TZP (Piperacilin-tazobactam), CAZ (ceftazidim); Cephem/Cephalosporin IV: FEP (cefepime); Aminoglycoside: GM (gentamicin), NN (tobramycin); Sulfonamide: SCP (cefoperazone/sulbactam).
