# Supplementary material for: Plant-expressed pyocins for control of Pseudomonas aeruginosa
Source: PLoS One. 2017 Oct 3;12(10):e0185782. doi: 10.1371/journal.pone.0185782 (PMC5626474; doi:10.1371/journal.pone.0185782)
Supplement: S1 Text — (PDF) [file pone.0185782.s002.pdf]

S1 text. Purification of plant-produced pyocins.

Pyocin S5 extraction and purification

A small portion of frozen leaf tissue was homogenized with chilled mortar and pestle in liquid nitrogen. Prepared powder was mixed with cold extraction buffer (50 mM HEPES, 10 mM CH_3COOK, 5 mM $Mg(CH_3COO)_2$, 2 mM DTT, 1 mM EDTA, pH 5.0) at a ratio of 1 g of plant material to 5 ml of buffer. The crude extract kept on ice for 15-20 min. Cell debris were removed by centrifugation at 3220 *g,* at 4 °C for 20 min. Pellets were discarded and the supernatant was filtered by passing solution through membrane filters (pore sizes 5 µm and 0.22 µm). The pH of solution adjusted to 9.4 and formed precipitate removed by centrifugation at 3220 *g,* at 4 °C for 10 min. The supernatant taken as total soluble protein and applied for purification in two steps.

At the first purification step, the chromatography column was filled with Q sepharose FF resin (GE Healthcare Life Sciences, Uppsala, Sweden) and pre-equilibrated with cold buffer (50 mM Tris, pH 9.4). Protein solution was loaded to column and unbounded Pyocin S5 was washed with equilibration buffer. Collected protein fraction was replaced to the diafiltrating concentrator (10 kDa) and centrifuged at 3220 *g* until volume of solution decreased 10-12 folds. Concentrate was diluted up to primary volume with extraction buffer and subjected to final purification step using CM sepharose FF resin (GE Healthcare Life Sciences, Uppsala, Sweden). Chromatography media was pre-equilibrated with cold buffer (50 mM HEPES, 10 mM CH_3COOK, 5 mM $Mg(CH_3COO)_2$, 2 mM DTT, 1 mM EDTA, pH 5.0). Protein solution was loaded to the column and CM sepharose-bounded protein fraction was eluted by linear gradient of cold buffer additionally containing 300 mM of NaCl. Collected Pyocin S5 was freeze-dried and applied for analysis.

Pyocin M extraction and purification

A small portion of frozen leaf tissue was homogenized with chilled mortar and pestle in liquid nitrogen. Prepared powder was mixed with cold extraction buffer (50 mM Tris, 5 mM CH_3COONa, pH 5.0) at a ratio of 1 g of plant material to 5 ml of buffer. The crude extract was kept on ice for 15-20 min. Cell debris were removed by centrifugation at 3220 *g* at 4 °C for 20 min. Pellets were discarded and the supernatant was filtered by passing solution through membrane filters (pore sizes 5 µm and 0.22 µm). $(NH_4)_2SO_4$ was added to the solution until the concentration of ammonium sulphate attained 1M. The pH of solution adjusted to 7.0, and protein solution applied for purification in two steps.

At the first purification step, the chromatography column was filled with Phenyl sepharose FF resin (GE Healthcare Life Sciences, Uppsala, Sweden) and pre-equilibrated with cold buffer (50 mM Tris, 1.0 M $(NH_4)_2SO_4$, 5 mM CH_3COONa, pH 7.0). Protein solution was loaded to column and the Phenyl sepharose-bounded protein fraction was eluted by washing with elution buffer (20 mM Tris, 0.6 M $(NH_4)_2SO_4$, pH 8.5). Collected protein fraction replaced to the diafiltrating concentrator (10 kDa) and centrifuged at 3220 *g* until the volume of protein solution decreased 20-25 folds. Concentrate was diluted up to primary volume with 20 mM Tris buffer (pH 8.5) and subjected to the final purification step using Q sepharose FF resin (GE Healthcare Life Sciences, Uppsala, Sweden). Chromatography media was pre-equilibrated with cold 20 mM Tris buffer (pH 8.5). Protein solution was loaded to column and Q sepharose-unbounded protein fraction was washed with equilibration buffer. Collected Pyocin M was freeze-dried and applied for analysis.

Pyocin M4 extraction and purification

A small portion of frozen leaf tissue was homogenized with chilled mortar and pestle in liquid nitrogen. Prepared powder was mixed with cold extraction buffer (50 mM Tris, 5 mM CH_3COONa, pH 5.0) at a ratio

of 1 g of plant material to 5 ml of buffer. The crude extract kept on ice for 15-20 min. Cell debris were removed by centrifugation at 3220 *g,* at 4 °C for 20 min. Pellets were discarded and supernatant filtered by passing solution through membrane filters (pore sizes 5 µm and 0.22 µm). $(NH_4)_2SO_4$ was added to the solution until the concentration of ammonium sulphate attained 1 M. The pH of solution adjusted to 7.0, and protein solution applied for purification in two steps.

At the first purification step, the chromatography column was filled with Phenyl sepharose FF resin (GE Healthcare Life Sciences, Uppsala, Sweden) and pre-equilibrated with cold buffer (50 mM Tris, 1.0 M $(NH_4)_2SO_4$, 5 mM CH_3COONa, pH 7.0). Protein solution was loaded to column, and Pyocin M4 eluted by washing with elution buffer (50 mM Tris, 0.4 M $(NH_4)_2SO_4$, 5 mM CH_3COONa, pH 7.0). Collected protein fraction replaced to the diafiltrating concentrator (10 kDa) and then centrifuged at 3220 *g* until volume of protein solution decreased 20-25 folds. Concentrate was diluted up to a primary volume with 50 mM Tris/5 mM CH_3COONa buffer (pH 8.5) and subjected to the final purification step using Q sepharose FF resin (GE Healthcare Life Sciences, Uppsala, Sweden). Chromatography media was pre-equilibrated with cold buffer (50 mM Tris, 5 mM CH_3COONa, pH 8.5). Protein solution was loaded to column and Q sepharose-bounded protein fraction was eluted by washing with elution buffer (50 mM Tris, 5 mM CH_3COONa, pH 5.0). Collected Pyocin M4 was freeze-dried and applied for analysis.

Pyocin L1 and L2 extraction and purification

Plant material was homogenized with chilled mortar and pestle using liquid nitrogen. Crude protein extract was mixed with cold extraction buffer containing 50 mM HEPES, 10 mM CH_3COOK , 5 mM $Mg(CH_3COO)_2$, 2 mM DTT, 1 mM EDTA (pH 5.0) at a ratio of 1 g of tissue to 5 ml of buffer. The suspension was kept on ice for 15 – 20 min and then centrifuged at 3220 *g*, at 4 °C for 30 min. The pellets were discarded and supernatant was filtered through membrane filters (pore sizes 5 µm and 0.22 µm).

Pyocins L1 and L2 were purified by two-step chromatography procedure. The first step consisted of hydrophobic interaction chromatography on Phenyl sepharose FF resin (GE Healthcare Life Sciences, Uppsala, Sweden). $(NH_4)_2SO_4$ was added to the crude protein extract until the concentration attained 1.0 M and the pH adjusted to 7.0. The supernatant was then loaded on a Phenyl sepharose column, which had been pre-equilibrated with cold extraction buffer containing 1.0 M $(NH_4)_2SO_4$ (pH 7.0). Pyocins L1 and L2 were eluted by washing with elution buffer (50 mM HEPES, 10 mM CH_3COOK , 5 mM $Mg(CH_3COO)_2$, 2 mM DTT, 1 mM EDTA, pH 7.0). The collected protein fraction was diluted with 50 mM HEPES, 10 mM CH_3COOK , 5 mM $Mg(CH_3COO)_2$, 2 mM DTT, 1 mM EDTA (pH 8.0) and then applied for the second purification step by anion exchange chromatography.

Q sepharose FF resin (GE Healthcare Life Sciences, Uppsala, Sweden) was pre-equilibrated with cold extraction buffer (pH 8.0). Protein sample was applied to a Q sepharose column. Pyocins L1 and L2 were collected in the flow-through fraction. The column was washed with the same buffer and the adsorbed fraction (contaminants) was then eluted from the column with elution buffer (extraction buffer containing 1M NaCl, pH 8.0).

Pyocin L3 extraction and purification

Plant material was homogenized with chilled mortar and pestle in liquid nitrogen. Crude protein extract was mixed with cold extraction buffer containing 50 mM HEPES, 10 mM CH_3COOK, 5 mM $Mg(CH_3COO)_2$, 2 mM DTT, 1 mM EDTA (pH 5.0) at a ratio of 1:5. The suspension was kept on ice for 15 -20 min and then centrifuged at 3220 x g, at 4 °C for 30 min. Cell debris were removed, and the supernatant was filtered through membrane filters (pore sizes 5 µm and 0.22 µm).

Pyocin L3 was purified by two-step chromatographic process. The first step consisted of hydrophobic interaction chromatography on Phenyl sepharose FF resin (GE Healthcare Life Sciences, Uppsala, Sweden). $(NH_4)2SO_4$ was added to the crude protein extract until the concentration attained 0.6 M and the pH adjusted to 7.0. The supernatant was then loaded on a Phenyl sepharose column, which had been pre-equilibrated with cold extraction buffer containing 0.6 M $(NH_4)_2SO_4$ (pH 7.0). Pyocin L3 was eluted by washing with elution buffer (50 mM HEPES, 10 mM CH_3COOK, 5 mM $Mg(CH_3COO)_2$, 2 mM DTT, 1 mM EDTA, pH 7.0). The collected protein fraction was diluted with 50 mM HEPES, 10 mM CH_3COOK, 5 mM $Mg(CH_3COO)_2$, 2 mM DTT, 1 mM EDTA (pH 8.0) and then applied for the second purification step by anion exchange chromatography. Q sepharose FF resin (GE Healthcare Life Sciences, Uppsala, Sweden) was pre-equilibrated with cold extraction buffer containing 50 mM NaCl (pH 8.0). Protein sample was applied to Q sepharose column. Pyocin L3 was collected in the flow-through fraction. The column was washed with the same buffer and the adsorbed fraction (contaminants) was eluted from the column with elution buffer (extraction buffer containing 0,5 M NaCl, pH 8.0).
