# Supplementary material for: Plant-expressed pyocins for control of Pseudomonas aeruginosa
Source: PLoS One. 2017 Oct 3;12(10):e0185782. doi: 10.1371/journal.pone.0185782 (PMC5626474; doi:10.1371/journal.pone.0185782)

S1 Fig. Determination of pyocin MIC by agar dilution method against *Pseudomonas aeruginosa* strain A19

Aliquots of melted CAA (1.5% agar) medium (final volume - 25 mL) adapted to 51 ^{0}C were supplemented with 0.1 mg mL^{-1} BSA and with appropriate amounts of pyocin stock solutions of different concentrations (ranging from 2400-9.4 ng mL^{-1} (concentrations lowered by a factor of 2)). Aliquots were poured into Petri plates.

P. aeruginosa strain A19 cultures were grown from single colony in CAA medium at 37 ^{0}C, 200 rpm for appr. 2 hours until OD_{595}=0.2 and diluted to 1*10^7 CFU mL^{-1}. 5 µL of bacteria suspension were applied in three replicates on each test plate.

Petri plates were incubated at 37 ^{0}C overnight. The determined MIC is a concentration of pyocin, where no confluent bacterial growth is observed.

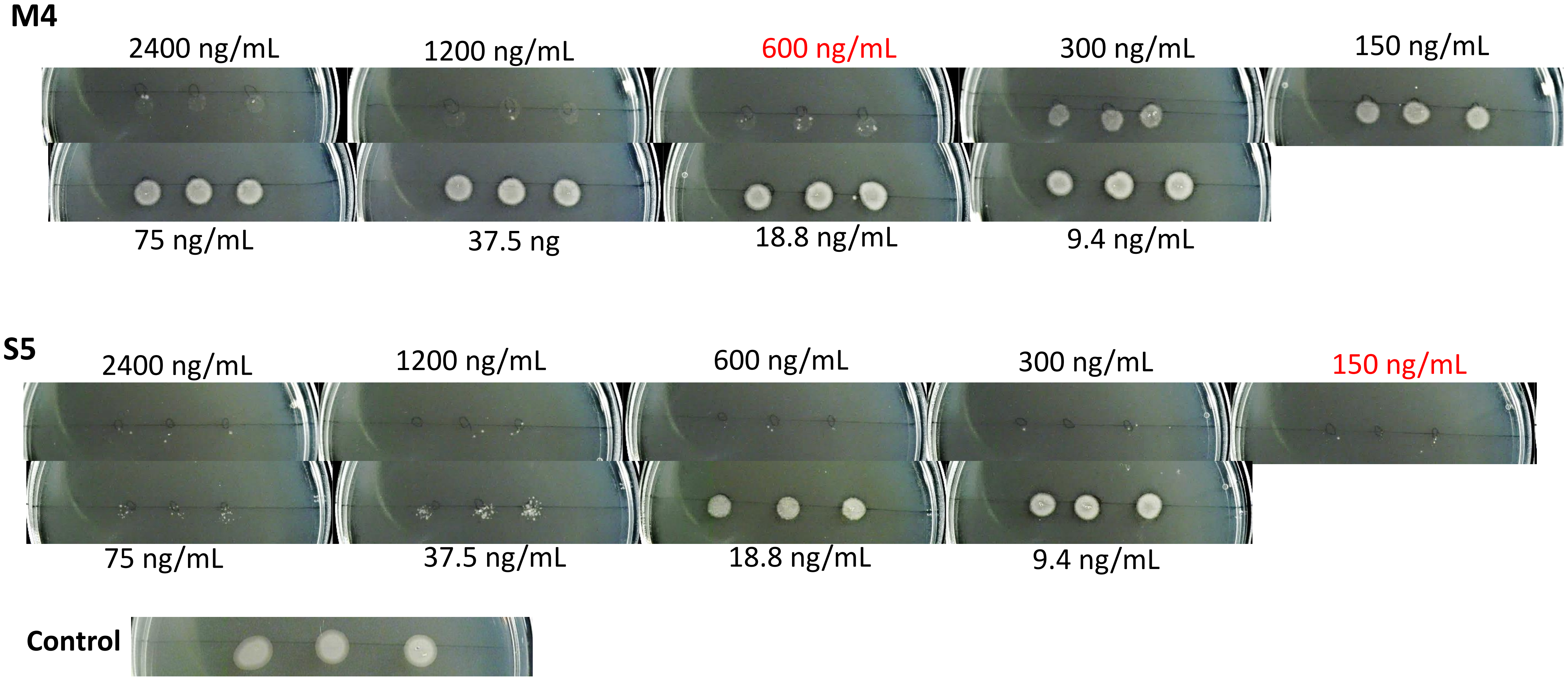

Supplement: S1 Fig — (PDF) [file pone.0185782.s003.pdf]
