# Supplementary material for: Plant-expressed pyocins for control of Pseudomonas aeruginosa
Source: PLoS One. 2017 Oct 3;12(10):e0185782. doi: 10.1371/journal.pone.0185782 (PMC5626474; doi:10.1371/journal.pone.0185782)
Supplement: S2 Fig — (PDF) [file pone.0185782.s004.pdf]

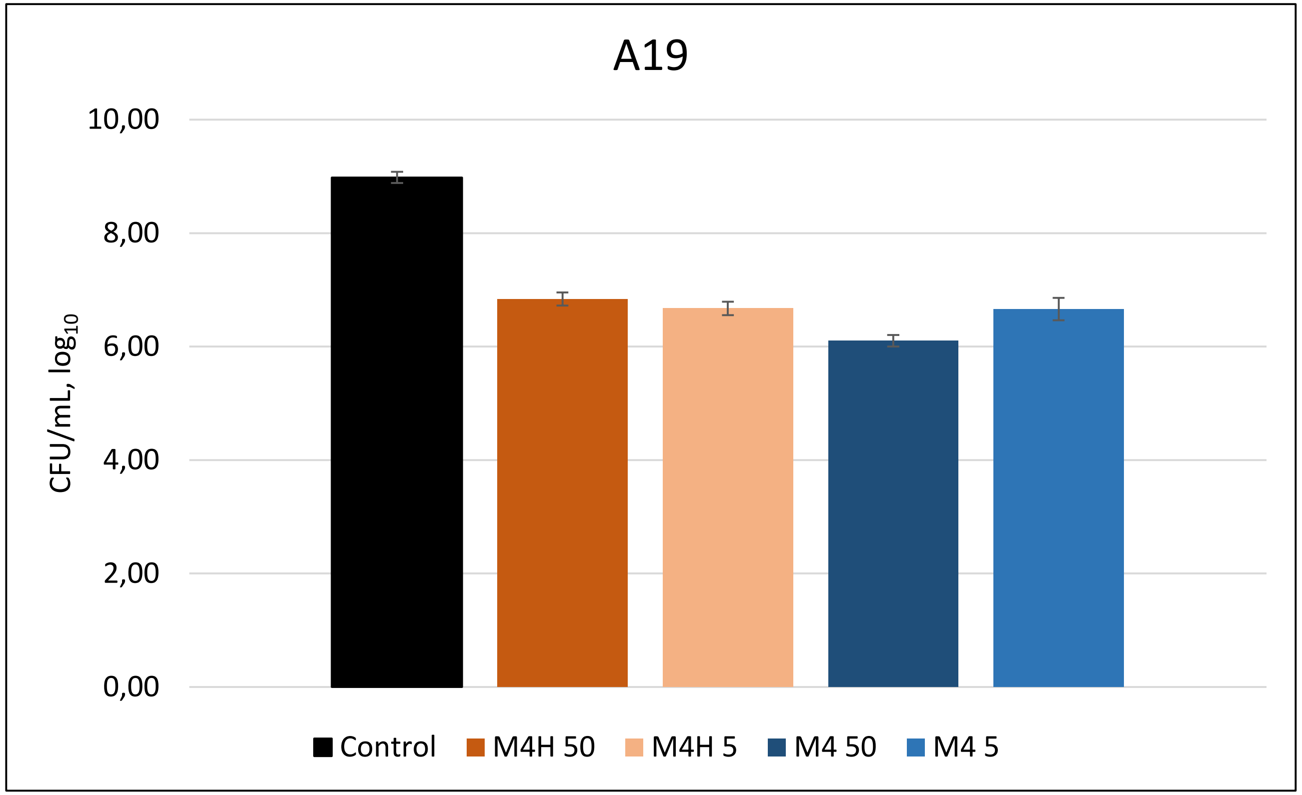

S2 Fig. PaeM4 and PaeM4H liquid culture killing assay. *P. aeruginosa* strain A19 was cultivated in CAA medium, treated by 5 or 50 µg mL^{-1} of pyocins and incubated with shaking for 6.5 hours. The antimicrobial activity of pyocins was evaluated by determining cell numbers of bacterial test culture. 10, 10^{-1}, 10^{-2}, 10^{-3}, 10^{-4}, 10^{-5} dilutions were made, plated on LB agar plates, incubated overnight at 37 °C and CFU calculated. Data are the mean ± SD of two independent experiments.
