# Supplementary material for: Plant-expressed pyocins for control of Pseudomonas aeruginosa
Source: PLoS One. 2017 Oct 3;12(10):e0185782. doi: 10.1371/journal.pone.0185782 (PMC5626474; doi:10.1371/journal.pone.0185782)
Supplement: S4 Fig — (PDF) [file pone.0185782.s006.pdf]

A

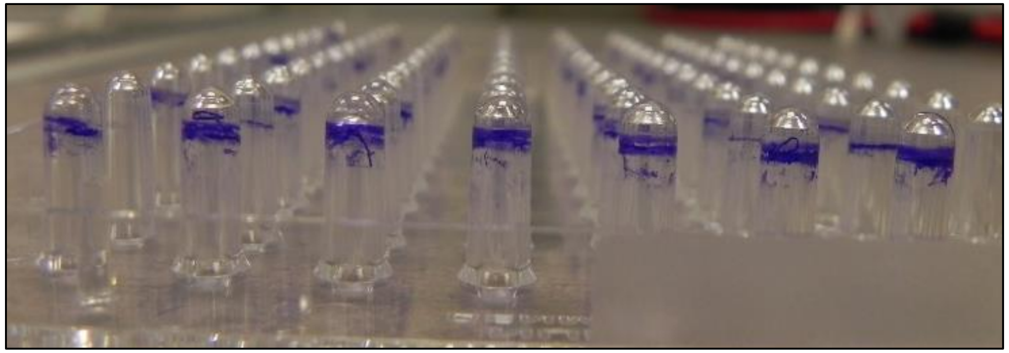

B

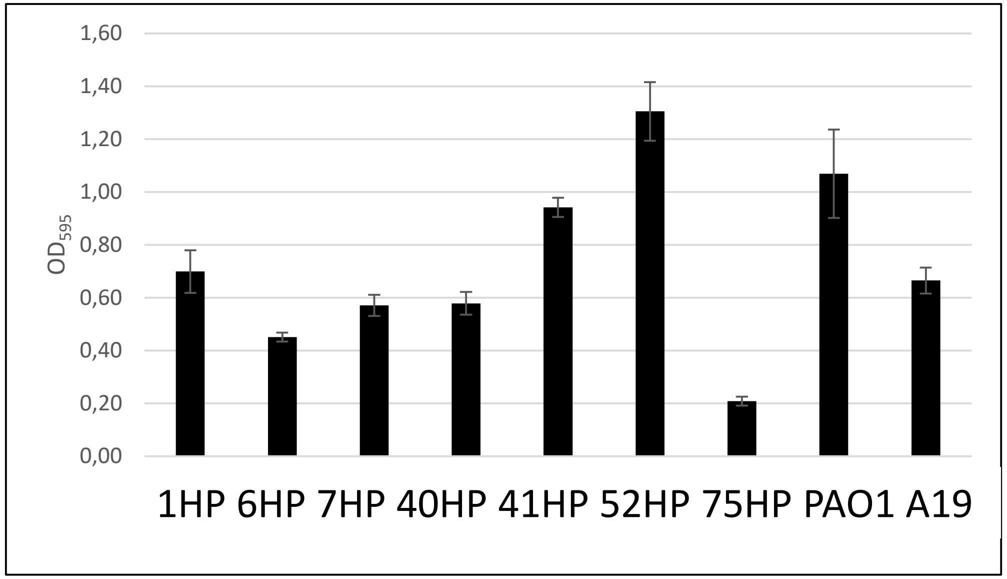

S4 Fig. *P. aeruginosa* biofilms.

A. Crystal violet–stained pegs after growing the biofilms of *P. aeruginosa* A19 for 20 h at 37 °C and washing three times. *P. aeruginosa* tends to form biofilms at liquid–air interface. The biofilm - forming bacteria are not eliminated by washing and stains blue with crystal violet.

B. The ability of different *P. aer*uginosa strains to form biofilm in CAA medium. To solubilize adsorbed crystal violet, pegs with stained biofilms were incubated in 95% ethanol for 15 min. The absorbance of ethanol was read at 595 nm. Bars represent standard deviation of absorbance of extracts from six pegs.
